# Revealing New Structural Insights from Surfactant Micelles through DLS, Microrheology and Raman Spectroscopy

**DOI:** 10.3390/ma8063754

**Published:** 2015-06-19

**Authors:** Samiul Amin, Steven Blake, Rachel C. Kennel, E. Neil Lewis

**Affiliations:** 1 Malvern Instruments, 7221 Lee Deforest Drive, Suite 300, Columbia, MD 21046, USA; E-Mails: steve.blake@malvern.com (S.B.); neil.lewis@malvern.com (E.N.L.); 2 Chemical and Biomolecular Engineering, University of Delaware, 150 Academy St., Newark, DE 19716, USA; E-Mail: rckennel@udel.edu

**Keywords:** wormlike micelles, microrheology, DLS, Raman Spectroscopy, branching, viscoelasticity

## Abstract

The correlation between molecular changes and microstructural evolution of rheological properties has been demonstrated for the first time in a mixed anionic/zwitterionic surfactant-based wormlike micellar system. Utilizing a novel combination of DLS-microrheology and Raman Spectroscopy, the effect of electrostatic screening on these properties of anionic (SLES) and zwitterionic (CapB) surfactant mixtures was studied by modulating the NaCl concentration. As Raman Spectroscopy delivers information about the molecular structure and DLS-microrheology characterizes viscoelastic properties, the combination of data delivered allows for a deeper understanding of the molecular changes underlying the viscoelastic ones. The high frequency viscoelastic response obtained through DLS-microrheology has shown the persistence of the Maxwell fluid response for low viscosity solutions at high NaCl concentrations. The intensity of the Raman band at 170 cm^−1^ exhibits very strong correlation with the viscosity variation. As this Raman band is assigned to hydrogen bonding, its variation with NaCl concentration additionally indicates differences in water structuring due to potential microstructural differences at low and high NaCl concentrations. The microstructural differences at low and high NaCl concentrations are further corroborated by persistence of a slow mode at the higher NaCl concentrations as seen through DLS measurements. The study illustrates the utility of the combined DLS, DLS-optical microrheology and Raman Spectroscopy in providing new molecular structural insights into the self-assembly process in complex fluids.

## 1. Introduction

Surfactant molecules can self-assemble to form vesicles or micelles, either spherical or cylindrical. The geometric constraints in surfactant packing and the bending elastic energy of their monolayers dictate the final morphology as hydrophobic and hydrophilic domains interact dynamically with each other and the surrounding medium to achieve the lowest energetic state [1]. For example, when surfactant packing constraints and/or headgroup interactions lead to exceptionally high curvature energy from micellar hemispherical endcaps, then cylindrical or wormlike micelles will begin to take shape [2]. The wormlike micelles can continue to grow and entangle (similar to polymer entanglement), exhibiting contour and persistence lengths of several microns and up to 20 nm, respectively, at equilibrium. The unique morphology and entanglement of wormlike micelles contribute high viscosity and viscoelasticity to the overall solution.

Due to their adaptability, wormlike micelles have been extensively studied in a large variety of systems: non-ionic surfactants [3,4,5], anionic surfactants [6,7,8], mixed anionic/cationic surfactants [9,10] and even in gemini surfactants [11,12]. The rich rheological and complex behavior of charged surfactants can be tuned by varying the salt concentration or pH. For many surfactant molecules, increasing the ionic strength of the solution gives rise to the formation of wormlike micelles as electrostatic screening of the charged polar head groups promotes cylindrical micellar elongation. At the macroscopic level, entanglement of these worms causes an initial increase in the viscosity of the bulk solution. However, a further increase of the ionic strength results in diminished viscosity as the entangled worms transition into branched micellar networks [13] or revert back to cylindrical or spherical micelles. Because there is a lack of characterization strategies that can correlate rheological behavior with microstructure at the molecular level, the exact mechanism underlying this behavior is not always characterized. The ultimate goal of obtaining mechanistic knowledge is to rationally design and efficiently fabricate new classes of materials that have desired properties and functionality. Ideally, this insight can be generated through a combination of characterization techniques that allows for the correlation of rheological, microstructural and molecular level structural changes.

Microrheology techniques involve optically tracking the motion of dispersed probe (or tracer) particles in a complex fluid to extract local and bulk rheological properties of the matrix. Analogous to mechanical rheometry, a stress is applied to the system by motion of the probe particle and the deformation (or strain) is measured through changes in the probe particle position.

Dynamic light scattering (DLS) microrheology is classified as a passive technique, whereby the colloidal probe particles, dispersed in the material under test, undergo thermal fluctuations in a system at thermodynamic equilibrium. The mean square displacement (MSD) of the probe particles with time is measured by DLS, and enables linear viscoelastic parameters for the complex fluid matrix to be extracted through a Generalized Stokes Einstein relationship [14]. DLS microrheology offers significant measurement advantages for complex fluids since it can measure over a much wider frequency range than conventional mechanical rheometry, which is fundamentally limited by inertia, and can access the very high frequencies required to measure the critical (short timescale) dynamics of such materials. These advantages are especially relevant for wormlike micelles, which can exhibit relaxation mechanisms spanning a wide range of timescales. Although highly relevant in the characterization of wormlike systems, the application of the technique has been relatively limited to date for such systems [15,16,17,18,19,20]. DLS microrheology can measure very small sample volumes (microliter-scale is possible), which enables the rheological characterization of materials not readily available in large volumes, e.g., protein-based formulations.

Raman Spectroscopy, in comparison, uses inelastic scattering to probe the molecular vibrations of the analyte, providing detailed molecular level structural information from the frequency and intensity of the resulting spectral bands. Raman data at wavenumbers less than 200 cm^−1^ are especially insightful with regards to microstructural/rheological changes as they are linked to intermolecular interaction effects such as hydrogen bonding and are also sensitive to changes in water structuring as it undergoes confinement into nanopores as a result of self-assembly of complex fluid systems.

In this study, we combine DLS-optical microrheology with Raman Spectroscopy to investigate the impact of ionic strength on molecular structure and associated changes in the microstructure and rheology of a mixture of anionic (sodium lauryl ether sulfate (SLES)), and zwitterionic (cocoamidopropyl betaine (CapB)), surfactants in aqueous solution. The simultaneous characterization of rheological and molecular structural information enables their correlation, providing insight into the molecular-level mechanisms that underlie changes in macro/physical properties. Alkylbetaines and alkylamidobetaines are a class of zwitterionic surfactants that are electroneutral in a wide pH range. They are utilized extensively with anionic surfactants such as SLES and sodium dodecyl sulfate as the base for many commercial bodywashes and shampoos. Although a few studies have characterized the formation of wormlike micelles formed in sodium dodecyl sulfate (SDS)/CapB system [21], to our knowledge, despite its commercial importance, there has been little characterization of the SLES/CapB surfactant mixture [22]. These limited scattering studies [21,22] on this system have however confirmed the transition to cylindrical and then wormlike micelles for this system at relatively low total surfactant concentrations. To the best of our knowledge, a systematic study on the effect of salt concentration on the evolution of the microstructure/rheology has not, however, been undertaken. This study provides the first detailed systematic characterization of the microstructure/rheology evolution in this specific wormlike system as a function of salt concentration and utilizes a combination of DLS, DLS-optical microrheology and Raman Spectroscopy to correlate microrheology, molecular structural changes and microstructure.

## 2. Results and Discussion

### 2.1. Dynamic Light Scattering

**Figure 1:**
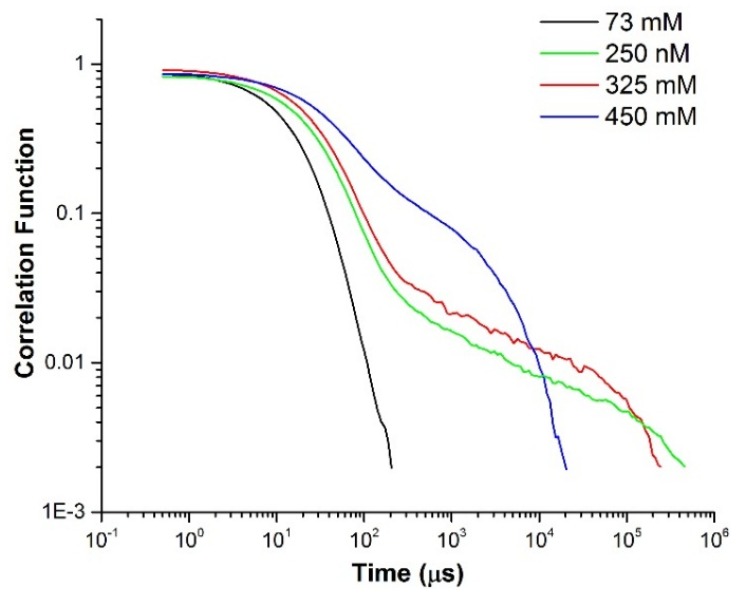
Correlograms illustrate a single decay mode during relaxation for the surfactant mixture of SLES : CapB (14% w/w : 2% w/w) containing 73 mM NaCl at 25 °C, while the other more viscous samples show a two-stage decay during relaxation.

Dynamic Light Scattering (DLS) measurements were carried out on separate SLES/CapB (14% w/w : 2% w/w) solutions at different salt concentrations. Figure 1 illustrates the evolution of the correlation function with increasing NaCl concentration. For clarity of the figure, data is shown for four NaCl concentrations.

The correlation functions exhibit quite striking changes with increasing NaCl concentrations. At the lowest concentrations investigated (73 mM NaCl), the correlation function clearly exhibits a single decay mode and has a fast decay. With increasing NaCl concentration the overall decay time increases, but, more interestingly, the evolution of a slow mode is also observed. This evolution of a slow mode in the DLS correlation function has been reported [23] for other wormlike micelle forming systems as a function of salt concentration. As the NaCl concentration is increased further (e.g., at 450 mM NaCl), the overall decay becomes faster, but the presence of a slow mode still persists. The faster relaxation at these high NaCl concentrations is indicative of a microstructural change under these conditions. The diffusion coefficients were extracted out for SLES (14% w/w), CapB (2% w/w with 73 mM NaCl) and SLES/CapB (14% w/w : 2% w/w with 73 mM NaCl), and the results are presented in Table 1. It should be noted that the correlation function for all three of these exhibits a single mode decay. The SLES/CapB combination in presence of 73 mM NaCl has a much smaller diffusion coefficient than either the SLES or CapB (albeit in the presence of 73 mM NaCl). This is indicative of a significant change in size of the micelles compared to just the SLES or CapB. The overall surfactant concentrations utilized in this study was 450 mM, which is far above the CMC and the sphere-to-rod transition concentrations reported for this system [21,22] (albeit at different SLES/CapB ratios). For our purposes, the weight percentage ratio of 14:2 for the surfactants was chosen due to the mixture remaining homogeneous at high salt concentrations.

### 2.2. DLS-Optical Microrheology

**Figure 2:**
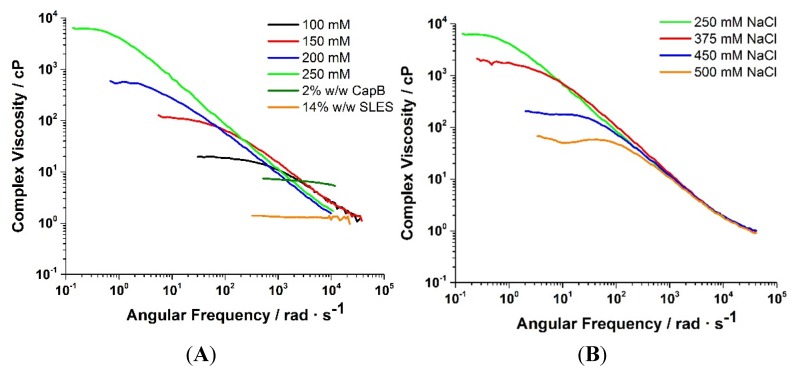
(**A**) Viscosity enhancement and evolution of non-Newtonian behavior for a mixed SLES/CapB system with changing salt concentration; (**B**) Decreasing viscosity while maintaining non-Newtonian behavior after peaking at 250 mM NaCl. 250 mM NaCl is shown in both figures as a point of reference.

Microrheology measurements were initially carried out on separate SLES (14% w/w) and CapB (2% w/w) solutions, as well as a mixture of the two in the presence of 100 mM NaCl solution. The complex viscosity of the surfactant samples was assessed by microrheological measurements. As illustrated in Figure 2, the 14% w/w SLES (no CapB) sample exhibits Newtonian behavior (viscosity independent of angular frequency) with a viscosity similar to water. This behavior, seen with a sample whose concentration is above the critical micelle concentration, can be indicative of the presence of spherical micelles. The sample containing 2% w/w CapB (no SLES) not only shows higher viscosity than the 14% w/w SLES sample but exhibits slight non-Newtonian behavior. This may be due to the presence of 73 mM NaCl in the commercial starting material. The DLS-reported diffusion coefficients for this sample were also lower than the SLES system. This maybe indicative of more rodlike or cylindrical micelles forming under these conditions. Betaines have been seen to form wormlike micelles in the presence of salts [2,3,6,9,12,13,14,17,21,22,23,24,25]. The samples containing both SLES/CapB (14% w/w and 2% w/w, respectively) exhibited increasing viscosity and non-Newtonian (shear-thinning) behavior as the NaCl concentration was increased to 250 mM.

**Table 1:** Diffusion coefficients associated with surfactant mixtures.

Samples	Diffusion Coefficient (µm^2^/s)
SLES (14% w/w with no NaCl)	165
CapB (2% w/w with 73 mM NaCl)	94.1
SLES/CapB (14% w/w :2% w/w with 73 mM NaCl	46.6

**Figure 3:**
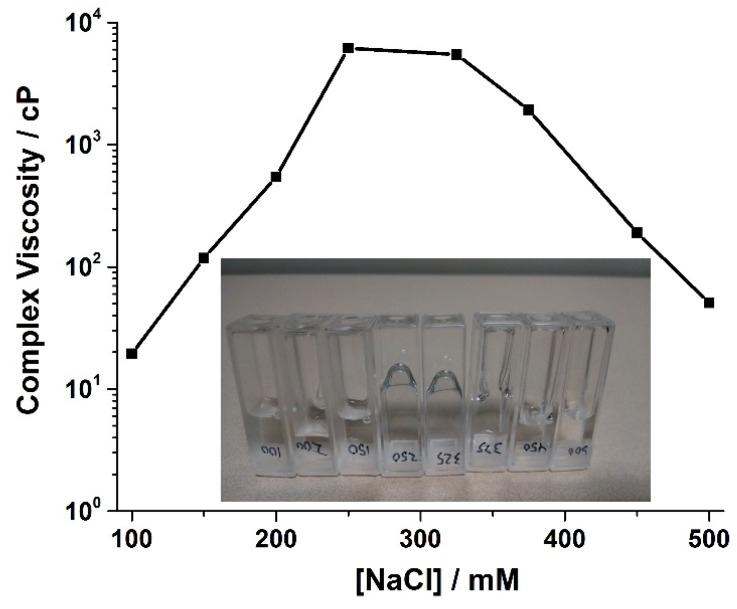
Viscosity exhibiting a maximum at 250 mM NaCl concentration.

Figure 2B shows that beyond 250 mM NaCl, the viscosity decreased monotonically, which was validated by inverting the samples and visually inspecting them (Figure 3). The non-Newtonian behavior, however, was maintained even at the highest NaCl concentration (500 mM). The decrease in viscosity in these systems at high NaCl concentrations is consistent with the DLS correlation functions having faster decays at higher NaCl concentrations. These behaviors will be discussed independently, starting with viscosity.

The decrease in viscosity in wormlike micellar systems with increasing salt concentration has been associated with different mechanisms: 1) shortening of the micelles or 2) the formation of branches [10]. To specifically determine which of the two mechanisms in the underlying microstructure is responsible for the decrease in viscosity, the frequency response of the viscoelastic moduli (G′ and G″) was analyzed in detail. As relaxation mechanisms tend to span a wide frequency range into the very highest (*i.e.*, short time scales), DLS-microrheology is an ideal tool to elucidate the underlying microstructural transitions in this system.

#### Viscoelastic Response and Evolution of Maxwellian Response

The rheological response in wormlike micelles is usually described in terms of the Cates model [26]. According to this model, there are two dominant relaxation mechanisms in entangled wormlike micelles: 1) reptation; and 2) breaking and recombination. When breaking and recombination is much faster than reptation, the solution behaves as a Maxwellian fluid with a single relaxation time given by [26]:

(1) tR=(τbτrep)12

Where *τ_b_* is the breaking/recombination time and *τ_rep_* is the reptation time. Under these conditions, the elastic modulus G′ and the viscous modulus G″ can be described by:

(2) $$G\prime =\frac{G_{p}\omega^{2}t_{R}^{2}}{1+\omega^{2}t_{R}^{2}}$$

(3) $$G\prime \prime =\frac{G_{p}\omega t_{R}}{1+\omega^{2}t_{R}^{2}}$$

Here, *G_p_* is the plateau modulus. The frequency response of the viscoelastic moduli (G′ and G″) for the SLES/CapB aqueous mixture at a fixed surfactant concentration and varying NaCl concentrations was obtained and analyzed for the expected Mawellian response from the entangled wormlike micelles.

**Figure 4:**
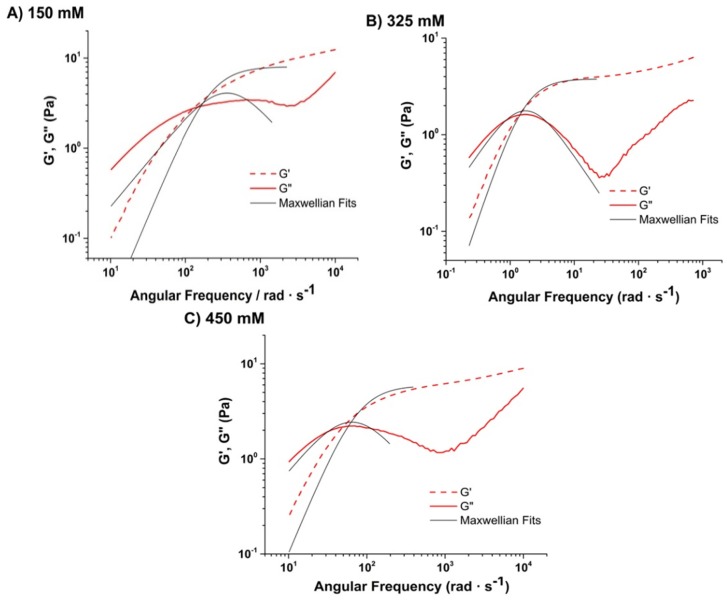
Evolution of frequency response of G′, G″ of SLES/CapB with increasing NaCl concentration: (**A**) 150 mM; (**B**) 325 mM and (**C**) 450 mM. Solid black lines are fits to the Maxwell model.

The response of G′ and G″ over the investigated frequencies exhibits trends seen in other ionic wormlike micelles where micelle growth is promoted through salt addition and screening of charges between headgroups [27,28]. Figure 4 illustrates the frequency response of G′ and G″ as a function of NaCl concentration. At low NaCl concentrations (150 mM), G′ does not exhibit a plateau and G″ does not exhibit a clear peak. As the NaCl concentration is increased further, G’ exhibits a clearer plateau and G” exhibits a more prominent peak, more typical of a Maxwellian fluid. In addition, the G′, G″ cross-over shifts to lower frequencies, which indicates much longer relaxation times. As NaCl concentration is increased further, this type of response is still maintained although the G′, G″ cross-over shifts to higher frequencies, indicating shorter relaxation times. Maxwell fits to the data are indicated in Figure 4. Although a perfect Maxwellian response was not recovered for the NaCl concentrations investigated, both the 325 mM and 450 mM NaCl concentration samples do exhibit closer fits than the 150 mM sample. The fit to the 325 mM data is in fact a reasonably good fit and then the fit becomes slightly poorer at the higher salt concentration; however, even then, the fit is still much better than that obtained at the lower (e.g., 150 mM) salt concentration. The lack of recovering a perfect Maxwell fit to the data could be attributed to potentially missing the NaCl concentration range where the exact required conditions exists.

The viscoelastic response as a function of salt concentration seen in Figure 4 is representative of what is seen in many ionic wormlike micelle systems as the micelles elongate and entangle. The difference in behavior at high salt concentration is worth a more detailed discussion though. At 450 and 500 mM NaCl, the samples exhibit relatively low viscosity values, similar to that seen at 150 mM NaCl concentration. However, the high salt samples exhibit more Maxwellian type behavior, whereas the low salt sample does not. This indicates that the low viscosity values seen for both low and high salt concentration samples potentially result from quite different underlying microstructure and relaxation dynamics.

Generally for wormlike micelles, the decrease in viscosity at high salt concentrations is attributed to either: 1) a transition to a branched micellar network; or 2) a reversal to short cylindrical micelles. Under both of these conditions, relaxation times decrease, with a corresponding change in zero shear viscosity, as was seen. In terms of Maxwellian behavior, however, theoretical and experimental work [29,30,31] done on relaxation dynamics in branching, indicate that speed of reptation increases with increasing micellar connections. In addition, a reversal to smaller micelles would also speed up reptation due to a reduction of contour length. Both of these scenarios would manifest in a deviation from Maxwellian behavior, which was not seen at high salt concentrations.

Instead the behavior for these samples that exhibit low viscosity with Maxwellian response at high salt concentration, could be characteristic of a moderately connected or branched network where, although reptation is sped up by the sliding of connections, it is still significantly longer than breaking/recombination times, due to the continued presence of topological constraints caused by entanglement. Given Maxwellian behavior for the high salt samples, it is difficult to ascertain the exact mechanism of viscosity reduction using only rheological data. To probe further into the underlying molecular level structural changes, Raman Spectroscopy was carried out on the SLES/CapB sample at different NaCl concentrations.

### 2.3. Raman Spectroscopy: Molecular Structural Changes

Raman Spectroscopy can reveal many supramolecular aspects of a complex mixture by probing reversible intermolecular interactions of its self-assembling constituents. This information is especially useful in analyzing changes in phase behavior of complex fluids, and understanding aggregation and gelation in such systems.

**Figure 5:**
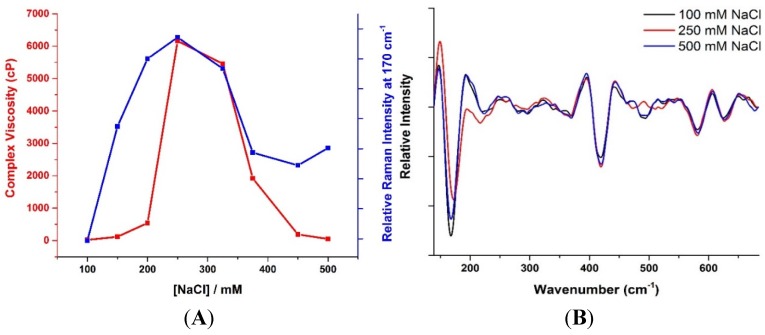
(**A**) Complex viscosity (red) and Raman intensity at 170 cm^−1^ (blue) of the SLES/CapB aqueous mixtures as a function of NaCl concentration. The Raman intensity is measured from the residual peak after subtraction of the bulk water component. These plots correspond to changes in the dynamics of confined water in relation to changes in viscosity; (**B**) Second derivative Raman spectra depicting the changes in the degree of confinement of water in 100 (black), 250 (red) and 450 mM NaCl (blue) surfactant solutions. Subtraction of the bulk water enables the bound water behavior to be isolated.

One way to gain mechanistic insight from these rheological changes is to monitor changes in water dynamics as a system undergoes gelation and self-assembly. The water dynamics can be determined through a number of different bands such as: 1) the low frequency bands at 60 cm^−1^ or 170 cm^−1^, which correspond to stretching and bending modes of hydrogen bonds between water molecules [32]; 2) the water bending band near 1645 cm^−1^; 3) combination of bending and librational bands near 2100 cm^−1^; and 4) the O-H stretching vibration band between 2800 and 3600 cm^−1^. Previous studies on gelation and self-assembly have focused on the O-H stretching band between 3200 and 3600 cm^−1^, and have primarily followed the change in dynamics of water, both bulk and bound forms. A recent study on agarose [33] monitored changes in dynamics of bound water through the band at 170 cm^−1^, and established a clear link between the evolving rheology in the system and water structuring in the polymer network. In this study, we continue to probe the degree of confinement of water in this self-assembling, worm-like system by monitoring changes in the intensity of this band. Figure 5 illustrates the variation of viscosity in conjunction with the Raman intensity at 170 cm^−1^. In general, the correlation between the viscosity and this peak intensity variation with NaCl concentration is consistent, with both showing a maximum at 250 mM NaCl and then dropping at higher NaCl concentrations. Although the viscosity drops to almost similar levels as exhibited at low salt concentrations, the low frequency Raman intensity does not completely recover at low NaCl concentrations. The intensity of this band at 170 cm^−1^ is linked to the damped stretching intermolecular modes of hydrogen bonded species and is sensitive to different structures of water [34,35,36]. The change in the intensity of the 170 cm^−1^ feature with initial increase in NaCl concentration is associated with enhanced water structuring and increased tetrahedral ordering of water [37,38,39]. From a microstructural perspective, this change may be attributed to an increase in the number of bound water molecules as the micelles grow from spherical to cylindrical. As the NaCl concentration is increased further, the viscosity and low frequency Raman intensity both drop, however the intensity does not return to the levels measured at low NaCl concentrations. This implies that the water molecules are becoming less structured, and that there is less correlated motion between water molecules, but not to the same levels as when the micellar system is unentangled or composed of short cylinders (at low NaCl concentration).

Given that the Maxwellian fluid response is still maintained at these high NaCl concentrations and that water structuring is still higher than observed in the unentangled regime, the possibility of a moderately branched network at high NaCl concentrations is more likely than a reversion to short micelles. Both microstructural scenarios would lead to a loss of viscosity, but a moderately branched network has been shown to maintain a Maxwellian response in other charged wormlike systems [28]. In the case of a moderately branched network, the microstructure is much more disordered and defect-prone. This would enhance unfavorable interactions of bound water with hydrophobic micellar cores and disrupt the confined water structure. Such an effect has been seen in other surfactant based systems where a transition from a lamellar phase to a sponge phase, which is more defect prone, led to a disruption of water structuring [35]. However, this disruption of water structuring probably has a smaller impact than that resulting from a reversal to small micelles, where the number of bound water molecules would be reduced significantly. Under conditions of modest branching, although there would be loss of water structuring for unfavorable interactions in the micellar connections, water structuring would still be maintained in the regions which did not have significant number of branch points. These regions would, in essence, exist as an entanglement of long wormlike micelles, and would exhibit continued Maxwellian response, the behavior seen at high NaCl concentrations. The dynamic light scattering measurements done on these systems, and as illustrated in Figure 1, also provide further evidence to this possible state of the underlying microstructure, as a persistence of a slow mode is seen in the higher salt concentration samples, whilst it is absent in the low salt concentration samples. The overall decay at these high salt concentrations (e.g., 450 mM) is, however, faster than seen at the intermediate salt concentrations (e.g., 250 mM), which exhibit the highest viscosity. These differences in the underlying microstructure would lead also to a difference in intensity of the Raman band at 170 cm^−1^ under high NaCl conditions compared to that seen at low NaCl conditions, which is also observed.

We note that, although the Raman intensities seen here exhibit a qualitative link with the viscosity changes, the response is weaker than that observed for the agarose system [29]. This is not unexpected, as the water in that system is quite significantly confined in the formation of the helical structure. Taken together though, the behavior of viscosity and Raman intensity seen in this study does provide insight into the underlying mechanism of the evolving microstructual changes. The clear link between Raman intensity at 170 cm^−1^ with branching and extent of branching may be obtainable through PGSE-NMR [29] or Cryo-TEM. Although beyond the scope of the present study, it is anticipated to be the focus of future work in the area.

## 3. Experimental Section

A combined DLS-Raman system (Helix, Malvern Instruments Ltd., Malvern, UK) has been used to obtain DLS (diffusion coefficient, nanoscale particle size) and Raman (structural information) data sequentially on a single sample. Microrheological measurements (viscosity and viscoelasticity) were performed with the same instrument but on a different sample. The Helix uses a proprietary non-invasive backscatter (NIBS) detector with dynamic (DLS), static (SLS) and electrophoretic (ELS) light scattering to measure the hydrodynamic radius of particles from 0.15 nm to 5 µm. Raman spectra are collected using 785 nm excitation (~280 mW) from 150 to 1925 cm^−1^ at 4 cm^−1^ resolution. DLS measurements and Raman Spectroscopy were performed on surfactant mixtures in water containing 14% w/w sodium lauryl monoether sulfate (SLES) (Chemservice Inc., West Chester, TX, USA), 2% w/w cocamidopropyl betaine (CapB) (The Lubrizol Corp., Wickliffe, WA, USA) and varying concentrations of sodium chloride (Sigma-Aldrich Corp., St. Louis, MO, USA), ranging from 73 to 500 mM. To perform the microrheological measurements, 900 nm polystyrene probe particles (Magsphere, Inc., Pasadena, CA, USA) were added to obtain a final concentration of 0.15% w/w in the surfactant mixtures and 73 mM NaCl due to commercial preparation of CapB. The tracer type and concentration were optimized to prevent interactions between the surfactant base and the tracer as well as to prevent tracer aggregation [24]. Sample aliquots (~20 µL) for Raman work were placed into a proprietary titanium cuvette with 120-µm thick quartz windows, and positioned in a temperature-stabilized sample holder, while for the microrheological work, 1-ml aliquots were placed into a disposable polystyrene cuvette and positioned in the same sample holder. The data described here were all collected at 25 °C.

## 4. Conclusions

The evolution of the rheological, microstructural and molecular structural changes in a wormlike micelle forming anionic/zwitterionic surfactant system has been investigated for the first time utilizing a novel combination of DLS, DLS-optical microrheology and Raman Spectroscopy. The viscoelastic response shows the persistence of nearly Maxwell fluid type characteristics for low viscosity solutions at high NaCl concentrations. Low frequency Raman intensity exhibits very good agreement with viscosity, as these parameters vary with salt concentration. The Raman intensity at high salt concentration does not recover to the values seen at low ones, indicating that there are microstructural and molecular structural differences at the extremes of concentration characterized in this study. This is further corroborated by persistence of a slow mode at the higher NaCl concentrations as seen through DLS measurements. The combined utilization of DLS, DLS-optical microrheology and Raman Spectroscopy has allowed the generation of new insights into the formulation-based rheological evolution in this wormlike micelle system, and can similarly be applied to investigate self-assembly in other complex fluid systems.
